# Genome-Wide CRISPR Screening Identifies DCK and CCNL1 as Genes That Contribute to Gemcitabine Resistance in Pancreatic Cancer

**DOI:** 10.3390/cancers14133152

**Published:** 2022-06-27

**Authors:** Hai Yang, Bin Liu, Dongxue Liu, Zhirong Yang, Shuman Zhang, Pengyan Xu, Yuming Xing, Isabella Kutschick, Susanne Pfeffer, Nathalie Britzen-Laurent, Robert Grützmann, Christian Pilarsky

**Affiliations:** 1 Department of Surgery, Universitätsklinikum Erlangen, Friedrich-Alexander-Universität Erlangen-Nürnberg (FAU), 91054 Erlangen, Germany; hai.yang@uk-erlangen.de (H.Y.); dongxue.liu@fau.de (D.L.); yangzhirong@stu.xmu.edu.cn (Z.Y.); shuman.zhang@extern.uk-erlangen.de (S.Z.); pengyan.xu@extern.uk-erlangen.de (P.X.); yuming.xing@extern.uk-erlangen.de (Y.X.); isabella.kutschick@uk-erlangen.de (I.K.); susanne.pfeffer@uk-erlangen.de (S.P.); nathalie.britzen-laurent@uk-erlangen.de (N.B.-L.); robert.gruetzmann@uk-erlangen.de (R.G.); 2 Cancer Research Center, Beijing Chest Hospital, Beijing Tuberculosis and Thoracic Tumor Research Institute, Capital Medical University, Beijing 101149, China; liubin@ccmu.edu.cn

**Keywords:** pancreatic cancer, CRISPR-Cas9, library screening, gemcitabine resistance, DCK, CCNL1

## Abstract

**Simple Summary:**

Pancreatic cancer is one of the most lethal cancers. Although complete surgical resection is the only curative treatment for pancreatic cancer, a late diagnosis is common and makes surgical treatment infeasible. Therefore, most patients receive chemotherapy to reduce the tumor burden. Gemcitabine has been the main chemotherapy for pancreatic cancer for over a decade; however, chemoresistance has emerged as a significant challenge to the efficacy of chemotherapy. In this study, we applied genome-wide CRISPR/Cas9 loss-of-function screening with gemcitabine treatment to identify DCK and CCNL1 as genes that contribute to gemcitabine resistance in pancreatic cancer and explored the mechanism of CCNL1-related gemcitabine resistance.

**Abstract:**

Pancreatic cancer is one of the most lethal cancers. Due to the difficulty of early diagnosis, most patients are diagnosed with metastasis or advanced-stage cancer, limiting the possibility of surgical treatment. Therefore, chemotherapy is applied to improve patient outcomes, and gemcitabine has been the primary chemotherapy drug for pancreatic cancer for over a decade. However, drug resistance poses a significant challenge to the efficacy of chemotherapy. The CRISPR/Cas9 (clustered regularly interspaced short palindromic repeats/CRISPR-associated protein 9) gene-editing system is a powerful tool, and researchers have developed CRISPR/Cas9 library screening as a means to identify the genes associated with specific phenotype changes. We performed genome-wide CRISPR/Cas9 knockout screening in the mouse pancreatic cancer cell line TB32047 with gemcitabine treatment and identified deoxycytidine kinase (DCK) and cyclin L1 (CCNL1) as the top hits. We knocked out DCK and CCNL1 in the TB32047 and PANC1 cell lines and confirmed that the loss of DCK or CCNL1 enhanced gemcitabine resistance in pancreatic cells. Many researchers have addressed the mechanism of DCK-related gemcitabine resistance; however, no study has focused on CCNL1 and gemcitabine resistance. Therefore, we explored the mechanism of CCNL1-related gemcitabine resistance and found that the loss of CCNL1 activates the ERK/AKT/STAT3 survival pathway, causing cell resistance to gemcitabine treatment.

## 1. Introduction

Pancreatic cancer is one of the most lethal cancers, with the lowest 5-year relative survival rate (10%). In 2021, the United States recorded approximately 60,430 new pancreatic cancer diagnoses and an estimated 48,220 deaths [1]. The only curative treatment for pancreatic cancer is complete surgical resection [2]. However, due to the lack of specific symptoms, a late diagnosis is common and makes surgical treatment impossible [3]. Therefore, most patients receive chemotherapy to reduce the tumor burden. Gemcitabine has been the main chemotherapy for pancreatic cancer for over a decade [4]. In recent years, combination chemotherapy has benefitted pancreatic cancer patients more than long-term gemcitabine treatment. Compared with gemcitabine alone, gemcitabine plus capecitabine and gemcitabine plus nab-paclitaxel improved progression-free survival (PFS), overall survival (OS), and response rates; gemcitabine-containing multi-drug combinations (GEMOXEL or cisplatin/epirubicin/5FU/gemcitabine) improved OS, PFS, and quality of life (QOL); gemcitabine plus platinum improved PFS and response rates, but not OS; and gemcitabine plus topoisomerase inhibitors increased toxicity but did not improve survival outcomes [5].

Although gemcitabine and other therapeutic drugs effectively treat pancreatic cancer, chemoresistance is a critical issue that substantially impacts the efficacy of chemotherapy. Compared with other chemotherapeutic drugs, pancreatic cancer cells are more resistant to gemcitabine. Therefore, most studies on chemoresistance have focused on gemcitabine. Gemcitabine resistance can be inherent or induced by drug treatment [6]. The reported drug-resistance mechanisms include drug transporters and the activation of enzymes and their targets. A variety of enzymes are involved in and regulate the transport, activation, and metabolism of gemcitabine. Multiple factors regulate the formation of drug resistance, such as the tumor microenvironment, the epithelial–mesenchymal transition, and microRNAs [7].

Researchers first discovered the CRISPR/Cas9 system in bacteria and archaea in 1987 [8], before applying it to gene editing in 2013 [9,10] and developing it into a powerful gene-editing tool [11]. Recently, researchers developed CRISPR/Cas9 library screening in cell culture and mouse models to identify the genes associated with cell proliferation, migration, invasion, and chemotherapy resistance [12,13,14].

We performed genome-wide CRISPR/Cas9 knockout screening in TB32047 cells, a murine cell line derived from a KPC model, to identify the genes that contribute to gemcitabine resistance. The top hits were sgRNAs for DCK and CCNL1. The inactivation of DCK and CCNL1 significantly increased the resistance to gemcitabine treatment in TB32047 and human-cell-line PANC1 cells. We then explored the regulatory mechanisms of CCNL1-related gemcitabine resistance, implicating the regulation of the ERK/AKT/STAT3 survival pathway.

## 2. Materials and Methods

### 2.1. Cell Culture

We used TB32047 and PANC1 cell lines. David Tuveson, CSHL (Cold Spring Harbor Laboratory, Cold Spring Harbor, NY, USA), kindly provided the primary mouse pancreatic cancer cell line TB32047, and we purchased the PANC1 cell line from ATCC (Cat. # CRL-1469, RRID: CVCL_0480, American Type Culture Collection, Manassas, VA, USA). We incubated all cell lines in a humidified incubator containing 5% CO_2_ at 37 °C and cultured TB32047 cells in DMEM (Dulbecco’s Modified Eagle’s Medium; Gibco, cat. # 31966-021) with 10% fetal bovine serum (FBS; Gibco, cat. # A3160801) and PANC1 cells in RPMI (Roswell Park Memorial Institute) medium 1640 (Gibco, cat. # 31870-025) with 10% FBS. We harvested all cells using 0.25% trypsin-EDTA (ethylenediaminetetraacetic acid) (Sigma, cat. # T4049-100ML). We generated the TB32047-Cas9 stable cell line by virally transducing lentiCas9-blast (Addgene, Cambridge, MA; cat. # 52962) into the cells and selecting them using 10 μg/mL of blasticidin (InvivoGen, cat. # ant-bl-1) for 3 days. We verified Cas9 expression by Western blot analysis.

### 2.2. Lentivirus Generation and Transduction

We generated lentivirus using HEK293TN cells (BioCat cat. # LV900A-1-GVO-SBI, RRID: CVCL_UL49) in T175 flasks. Briefly, we combined 9.2 µg pMDLg/pRRE plasmid (Addgene plasmid # 12251; http://n2t.net/addgene:12251 (accessed on 6 June 2022).; RRID: Addgene_12251), 4.6 µg pRSV-REV plasmid (Addgene plasmid # 12253; http://n2t.net/addgene:12253 (accessed on 6 June 2022); RRID: Addgene_12253), 4.6 µg pMD2.G plasmid (Addgene plasmid # 12259; http://n2t.net/addgene:12259 (accessed on 6 June 2022); RRID: Addgene_12259), and 13.8 µg mouse CRISPR knockout pooled library (GeCKO v2; Addgene # 1000000053) part A or B with lipofectamine 3000 (Thermo Fisher Scientific, cat. # L3000015) according to the manufacturer’s protocol and transfected the HEK293TN cells with the mixture. Twenty-four hours after the transfection, we collected the virus medium and centrifuged it at 2000 rpm for 10 min to remove cells and debris. Then, we filtered the supernatant with a 0.45 μm pore filter, aliquoted it into cryovials, and stored it at −80 °C. We transduced the TB32047-Cas9 cells with serial dilutions of the virus to determine an MOI of ~0.3. The pMDLg/pRRE, pRSV-REV, and pMD2.G were gifts from Didier Trono [15], and the mouse GeCKO v2 CRISPR knockout pooled library was a gift from Feng Zhang [16].

### 2.3. CRISPR Screening

We seeded 1 × 10^7^ TB32047-Cas9 cells into 2 T175 flasks. After 24 h, we transduced the cells with lentiviral mouse GeCKO v2 library part A or B at an MOI of 0.3 in the presence of 4 µg/mL polybrene for 24 h, then replaced the virus medium with fresh growth medium and cultured the cells for another 2 days. We selected the transduced cells with 10 μg/mL puromycin (InvivoGen, cat. # ant-pr-1) for 3 days and continued to culture them until we could harvest and seed enough cells for further gemcitabine treatment. We harvested 2.5 × 10^7^ cells for genomic DNA isolation and treated 1.1 × 10^7^ cells with gemcitabine at 50 nM (IC90 for TB32047 wild-type cells) for 3 days; then, we collected the surviving cells and cultured them until we had enough to repeat the gemcitabine treatment. After 5 rounds of gemcitabine treatment, we harvested the surviving cells for genome DNA isolation and performed NGS on the Illumina HiSeq 2500 platform with upwards of 5 × 10^7^ reads for each half-library in the Deep Sequencing Facility of TU Dresden. We counted and analyzed the fastq files using MAGeCK-VISPR software [17]. We counted and normalized the sequencing results of both half-libraries based on the results of NonTargetingControl-sgRNA, then combined and compared them using robust rank aggregation (RRA). We produced and transduced the lentivirus of both lentiviral mouse GeCKO v2 library part A and B into TB32047-Cas9 cells 3 times.

### 2.4. Genomic DNA Isolation and PCR Amplification

We extracted genomic DNA with NucleoSpin^®^ Blood XL (Machery Nagel, cat. # 740950.50) according to the manufacturer’s protocol. We performed the first-round PCR of NGS with 26 separate 100 μL redundant reactions, each containing 5 μg DNA, 50 μL Q5^®^ Hot Start High-Fidelity 2X Master Mix (NEB # M0494L), and 3 μL of a 10 μM solution of each universal illumine adapter tailed primer (P5 and P7). We structured the PCR amplification program as follows: step 1, 98 °C for 30 s; step 2, 98 °C for 10 s; step 3, 62 °C for 30 s; step 4, 72 °C for 30 s; repeat steps 2–4 25 times; step 5, 72 °C for 2 min. Subsequently, we indexed the PCR products with two indexing primers (P5 and P7) and sequenced the products as 75 bp single-end reads using NextSeq 500 (Illumina).

Briefly, we subjected purified PCR products with universal 5′ tails to a second PCR of 6–8 cycles using Phusion HF (NEB, Ipswich, MA, USA) and two indexing primers (P5 and P7). After indexing PCR, we purified the final libraries (1× Agencourt AMPure XP Beads, Beckman Coulter, Krefeld, Germany) and equimolarly pooled and sequenced them.

Universal illumine adapter tailed primer P5:

5′-ACACTCTTTCCCTACACGACGCTCTTCCGATCTNNNNNTCTTGTGGAAAGGACGAAACACCG-3′.

Universal illumine adapter tailed primer P7:

5′-GTGACTGGAGTTCAGACGTGTGCTCTTCCGATCTTCTACTATTCTTTCCCCTGCACTGT-3′.

Index primer P5:

5′-AATGATACGGCGACCACCGAGATCTACACiiiiiiii ACACTCTTTCCCTACACGACGCTCTTCCGATC*T-3′.

Index primer P7:

5′-CAAGCAGAAGACGGCATACGAGATiiiiiiii GTGACTGGAGTTCAGAC-GTGTGCTCTTCCGATCT-3′.

The CRISPR/Cas9 screening data are accessible in the ENA database under PRJEB51345.

### 2.5. CRISPR/Cas9 Gene-Editing Knockout System

We used the CRISPR/Cas9 gene-editing knockout system to knockout CCNL1 in the TB32047 and PANC1 cell lines and DCK in the TB32047 cell line. We used pSpCas9(BB)-2A-Puro (PX459) V2.0 (Addgene plasmid # 62988; http://n2t.net/addgene:62988 (accessed on 6 June 2022).; RRID: Addgene_62988) as a plasmid vector. This plasmid was a gift from Feng Zhang [18]. We synthesized sgRNAs through Eurofins. We designed the sgRNAs based on the human and mouse CRISPR knockout pooled library (Addgene # 1000000053, 1000000049) [16] or the online CRISPR/Cas9 target predictor (https://cctop.cos.uni-heidelberg.de/) (accessed on 6 June 2022) [19]. We constructed plasmids according to the protocol outlined in [18] and confirmed them by sequencing. We transfected the cells with constructed plasmids using lipofectamine 3000 transfection reagent (Invitrogen, cat. # L3000015, Waltham, MA, USA) for 72 h. Then, we selected the cells with 10 μg/mL puromycin for 3 days. We seeded the puromycin-selected cells into two 96-well plates to select single clones. We performed Western blot or RT–PCR to detect the knockout effect.

The sgRNAs used in this study were:

mm-NC-sg1—forward: 5′-CACCgACCAGGCGCGGACCGCACAT-3′, reverse: 5′-AAACATGTGCGGTCCGCGCCTGGTC-3′.

mm-NC-sg2—forward: 5′-CACCgAGGTCGTGGACCCGCATGTA-3′, reverse: 5′-AAACTACATGCGGGTCCACGACCTC-3′.

mm-CCNL1-sg1—forward: 5′-CACCgCTTACCAGGCAGTTTGAACC-3′, reverse: 5′-AAACGGTTCAAACTGCCTGGTAAGC-3′.

mm-CCNL1-sg2—forward: 5′-CACCgCAATGGGGCCGAGTTGGCAA-3′, reverse: 5′- AAACTTGCCAACTCGGCCCCATTGC-3′.

mm-DCK-sg—forward: 5′-CACCGGCTCGGATCCGGCTGAGAC-3′, reverse: 5′-AAACGTCTCAGCCGGATCCGAGCC-3′.

hu-NC-sg—forward: 5′-CACCgAAGGCGCGCGAATGTGGCAG-3′, reverse: 5′-AAACCTGCCACATTCGCGCGCCTTC-3′.

hu-CCNL1-sg1—forward: 5′-CACCgTAAGTGAAACTTCCGAGTAC-3′, reverse: 5′-AAACGTACTCGGAAGTTTCACTTAC-3′.

hu-CCNL1-sg2—forward: 5′-CACCgCAATGGGGACGAGTTGGCAA-3′, reverse: 5′-AAACTTGCCAACTCGTCCCCATTGC-3′.

hu-DCK-sg—forward: 5′-CACCgAGAGGTGCCTATCTTAACAC-3′, reverse: 5′-AAACGTGTTAAGATAGGCACCTCTC-3′.

### 2.6. Western Blot and Antibodies

We purchased anti-DCK antibodies from Abcam (Abcam Cat. # ab96599, RRID: AB_10680904) and Cas9 antibodies from Santa Cruz Biotechnology (Santa Cruz Biotechnology Cat. # sc-517386, RRID: AB_2800509). From Cell Signaling Technology, we purchased GAPDH (Cell Signaling Technology Cat. # 5174, RRID:AB_10622025); Phospho-p44/42 MAPK (Erk1/2, Thr202/Tyr204, D13.14.4E) (Cell Signaling Technology Cat. # 4370, RRID:AB_2315112); Phospho-Akt (Ser473) (Cell Signaling Technology Cat. # 4060, RRID:AB_2315049); Phospho-Stat3 (Tyr705) (Cell Signaling Technology Cat. # 9145, RRID:AB_2491009); p44/42 MAPK (Erk1/2) (Cell Signaling Technology Cat. # 4695, RRID:AB_390779); Akt(pan) (Cell Signaling Technology Cat. # 4691, RRID:AB_915783); Stat3 (Cell Signaling Technology Cat. # 12640, RRID:AB_2629499); anti-rabbit and anti-mouse IgG antibodies (Cell Signaling Technology Cat. # 7074, RRID:AB_2099233); and HRP-linked antibodies (Cell Signaling Technology Cat. # 7076, RRID:AB_330924). Briefly, we lysed the cells with RIPA buffer and collected the supernatant for gel electrophoresis. We transferred the proteins to a nitrocellulose membrane and detected the signals using an Amersham Imager 600 (Pittsburgh, PA, USA) with SignalFire Elite ECL Reagent (Cell Signaling Technology,12757S) or SignalFire ECL Reagent (Cell Signaling Technology, 6883). We individually performed all Western blot assays 3 times.

### 2.7. Quantitative RT–PCR

We performed RNA isolation using a NucleoSpin RNA Plus kit (Macherey-Nagel, #740984.250) and synthesized cDNA using a High-Capacity cDNA Reverse-Transcription Kit (Applied Biosystems, 00364942, Foster City, CA, USA). We used Power SYBR Green Master Mix (Applied Biosystems, 4367659) to conduct the quantitative RT–PCR and analyzed the results using the 2^−∆∆Ct^ method. We used beta-actin as a reference. We synthesized all primers through Eurofins Genomics (Ebersberg, Germany).

The qRT–PCR primers used in this study were:

mm-beta-actin—forward: 5′-GTGACGTTGACATCCGTAAAG-3′, reverse: 5′-GCCGGACTCATCGTACTCC-3′.

mm-CCNL1-qPCR—forward: 5′-GTCAAGCATCCCCATAAGATCA-3′, reverse: 5′- AAGAAACCAATGGGGCCGAG-3′.

hu-beta-actin—forward: 5′-CACCATTGGCAATGAGCGGTTC-3′, reverse: 5′-AGGTCTTTGCGGATGTCCACGT-3′.

hu-CCNL1-sg1-qPCR—forward: 5′-ATCGCCTGTACTCGGAAGTT-3′, reverse: 5′- GGGGCTTGGAGTCCTTTTTC-3′.

hu-CCNL1-sg2-qPCR—forward: 5′-TGTGAACGTAATCAAACCCTGG-3′, reverse: 5′-GAGTTGGCAACGGAATCTGA-3′.

### 2.8. Genome Mutation Confirmation

We used NucleoSpin^®^ Blood L (Machery Nagel, cat. # 740954.20) to isolate genome DNA from cell lines according to the manufacturer’s protocol. We amplified PCR productions for sequencing using Q5^®^ Hot Start High-Fidelity 2X Master Mix and cloned PCR fragments into pMiniT 2.0 using an NEB PCR Cloning Kit (New England Biolabs, cat. # E1203S). We sequenced plasmid DNA through Eurofins Genomics.

The sequencing PCR primers used in this study were:

mm-seq-CCNL1-sg1—forward: 5′-ACTCTGCGGTGAAAGAACCA-3′, reverse: 5′-AAGAGGAACTTACCAGGCAGT-3′.

mm-seq-CCNL1-sg2—forward: 5′-TGCTGTGGGGAAGTGGTTAG-3′, reverse: 5′-CCAAAATGGCATCAGCACCA-3′.

hu-seq-CCNL1-sg1—forward: 5′-GGCCTCATTCGACAGCTACT-3′, reverse: 5′-GTGATACTGAGATGCGGCGT-3′.

hu-seq-CCNL1-sg2—forward: 5′-GCTTGCATCTACCTTGCAGC-3′, reverse: 5′-GCATGTTGGCACAAACGAGT-3′.

### 2.9. Chemotherapeutic Drug Resistance Assay

We seeded the cells into a Corning^®^ 96-Well Black Polystyrene Microplate (3603) with 1 × 10^4^ cells per well for 24 h. Then, we added chemotherapeutic drugs to the wells. After 72 h of chemotherapeutic drug treatment, we stained the cells with DAPI (NucBlue; Biotium, cat. # 40046), imaged them using an EVOS FL Auto 2 imaging system (Invitrogen, AMAFD2000), and analyzed the images using HCS studio cell analysis software V2.0 (Thermo, SX000041A, Waltham, MA, USA). For TB32047 cells, the concentration assay of 5-FU ranged from 0.1 to 6.4 µM with 2-fold increments, and for PANC1 it ranged from 0.2 to 12.8 µM with 2-fold increments. For both TB32047 and PANC1 cells, the concentration assay of gemcitabine ranged from 1.25 to 80 nM with 2-fold increments. For TB32047 cells, the concentration assay of oxaliplatin ranged from 0.329 to 240 µM with 3-fold increments, and for PANC1 cells it ranged from 0.027 to 20 µM with 3-fold increments. We purchased chemotherapeutic drugs from the pharmacy of Universitätsklinikum Erlangen in ready-made solutions: gemcitabine—40 mg/mL, Hexal AG, Holzkirchen, Freistaat Bayern, Germany; oxaliplatin—50 mg/mL, Medac GmbH, Wedel, Niedersachsen, Germany; and 5-fluorouracil—5 mg/mL, Accord Medical Ltd., London, UK. We calculated the IC50 using GraphPad Version 8 (GraphPad Software, La Jolla, CA, USA).

### 2.10. Apoptosis Assay

We performed an apoptosis assay with APC Annexin V (Cat. # 550474, BD Pharmingen, San Diego, CA, USA) according to the manufacturer’s instructions. Briefly, we collected the cells (in the medium and adherent to the plates), washed them with cold PBS, resuspended them in the binding buffer, and stained them with APC Annexin V and PI for 15 min at room temperature. Then, we analyzed them with a BD Biosciences LSRII flow computer. We analyzed the flow cytometry results using FlowJo v10.8 software (BD Life Sciences, Ashland, OR, USA).

### 2.11. Statistical Analysis

We used MAGeCK-VISPR to rank and sort sgRNAs by *p*-value and/or FDR and GraphPad Version 8 and Microsoft Excel Version 2017 (Microsoft, Seattle, WA, USA) to perform statistical analyses. We used a *t*-test and one-way ANOVA for cell culture experiments. We considered *p*-values < 0.05 as statistically significant. We conducted the EC assay using GraphPad and carried out all assays individually at least three times (n ≥ 3).

## 3. Results

### 3.1. Genome-Wide CRISPR Screening Identifies DCK and CCNL1 as Genes Involved in Chemoresistance

**Figure 1:**
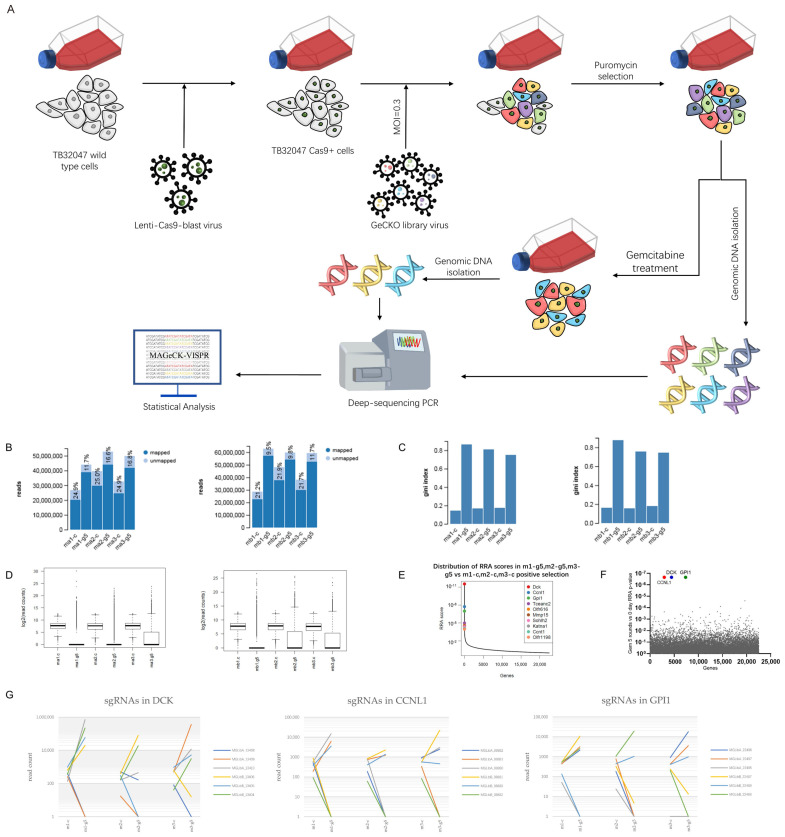
Genome-wide CRISPR screening in TB32047 cells revealed genes whose loss contributes to gemcitabine resistance. (**A**) positive-selection screening in TB32047-Cas9 cells. We treated the cells with 50 nM (IC90) gemcitabine for 3 days. After cell recovery, we repeated the gemcitabine treatment 4 more times and collected genomic DNA for deep sequencing. (**B**) Total reads, both mapped and unmapped. (**C**) Gini indices of the starting cells and screened cells. (**D**) Boxplot showing the sgRNA frequency distribution of the starting cells and screened cells. (**E**) Comparison of robust rank aggregation (RRA) score distribution indicated that DCK, CCNL1, and GPI1 were the top hits. (**F**) Identification of top candidate genes using RRA *p*-value analysis. (**G**) Distribution of sgRNA read counts (normalized) of DCK, CCNL1, and GPI1 in starting cells and screened cells.

We conducted positive-selection genome-wide CRISPR screening to identify the essential and/or contributory genes involved in resistance to gemcitabine, the current frontline chemotherapeutic agent. We transduced the full GeCKO libraries into the cells, harvested a portion of the library cells as our control samples, then treated the cells with 50 nM (approximate IC90) of gemcitabine for 3 days. We collected and cultivated the surviving cells and re-treated them with 50 nM of gemcitabine. After five rounds of gemcitabine treatment, we harvested the genomic DNA and performed next-generation sequencing. We counted and analyzed the fastq files using MAGeCK-VISPR software. We individually performed library transduction and gemcitabine treatment three times. We mapped approximately 80% of the reads to the sgRNA library. As a measurement of inequality, the GINI index differed substantially between the control groups and the treatment groups, indicating a selection event (Figure 1A–D).

In the gene ranking list, 1094 genes had a positive *p*-value below 0.05. However, only 16 had a log2-fold change above 0, and three of these had a positive false-discovery rate (FDR) below 0.05. The three most enriched genes in the CRISPR screen were DCK, CCNL1, and Gpi1, the loss of which causes resistance to gemcitabine treatment (Figure 1E–G, Table S1).

### 3.2. Loss of DCK and CCNL1 Enhances Resistance to Gemcitabine Treatment in TB32047 and PANC1 Cells

Next, we validated the top-ranking genes from the screening results. We generated the DCK-knockout cell lines using the CRISPR/Cas9 gene-editing system and detected the DCK-knockout effect by performing Western blot analysis. The DCK-negative cells were completely resistant to 80 nM gemcitabine (Figure S1). Compared with the wild-type (WT) and negative control (NC) cells, the DCK-negative cells were more resistant to gemcitabine treatment.

**Figure 2:**
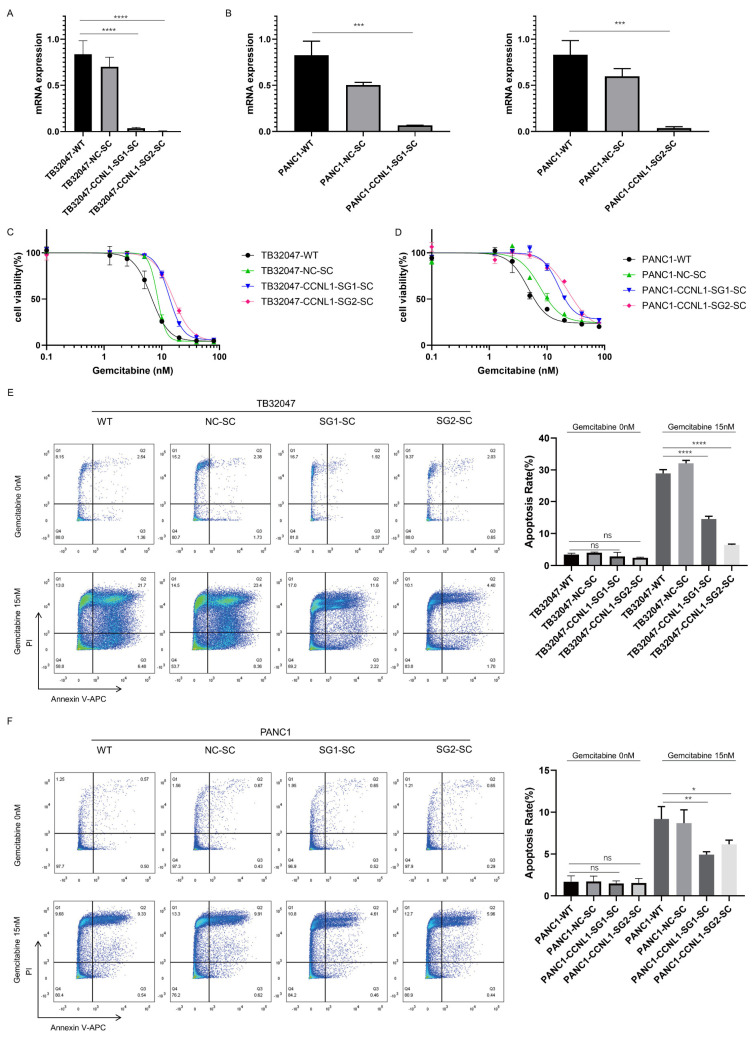
Knockout of CCNL1 increased gemcitabine resistance in TB32047 and PANC1 cells. (**A**,**B**) qRT–PCR results showing that CCNL1 was knocked out in TB32047-SG1-SC, TB32047-SG2-SC, PANC1-SG1-SC, and PANC1-SG2-SC. Data are presented as means of three independent experiments. *** *p*-value < 0.001, **** *p*-value < 0.0001 by one-way ANOVA. (**C**,**D**) Gemcitabine dose–response curves for TB32047 and PANC1 cells. (**E**,**F**) Flow-cytometry-based apoptosis analysis of TB32047 and PANC1 cells treated with 15 nM gemcitabine after 72 h. Data are presented as means of three independent experiments. * *p*-value < 0.05, ** *p*-value < 0.01, *** *p*-value < 0.001, **** *p*-value < 0.0001 by one-way ANOVA. CCNL1-negative cells were more resistant to gemcitabine than WT and NC cells.

We also generated CCNL1-knockout cell lines using the CRISPR/Cas9 gene-editing system. We used qRT–PCR and sequencing to detect the knockout of CCNL1 (Figure 2A,B and Figure S2, Table 1). The gemcitabine IC50 value for TB32047, PANC1 WT, and NC cells was around 5 nM, the value for TB32047 CCNL1-negative cells was nearly double that for WT and NC cells, and the value for PANC1 CCNL1-negative cells was around 20 nM (Figure 2C,D, Table 2). Without gemcitabine treatment, a flow cytometry analysis showed no significant difference in apoptosis between WT, NC, and CCNL1-negative cells. After 72 h of gemcitabine treatment, compared to WT and NC cells, both early and late apoptosis was reduced in TB32047 CCNL1-negative cells, and late apoptosis was reduced in PANC1 CCNL1-negative cells (Figure 2E,F). Compared with WT and NC cells, CCNL1-negative cells were more resistant to gemcitabine treatment.

### 3.3. Knockout of CCNL1 Promotes the Phosphorylation of Erk, Akt, and Stat3 in TB32047 and PANC1 Cells

**Figure 3:**
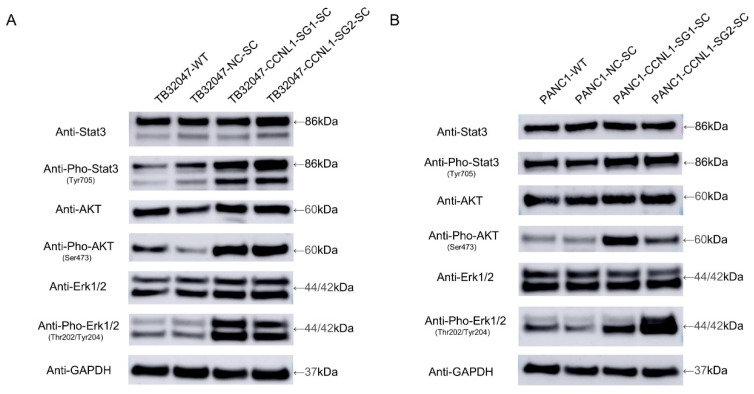
Knockout of CCNL1 promotes the phosphorylation of Erk, Akt, and Stat3. (**A**,**B**) Phosphorylation of Erk, Akt, and Stat3 is upregulated in CCNL1-knockout TB32047 and PANC1 cells.

We observed an upregulated phosphorylation of Erk (extracellular-signal-regulated kinase), Akt (protein kinase B (PKB)), and Stat3 (signal transducer and activator of transcription 3) in CCNL1-knockout TB32047 and PANC1 cells, whereas the expression levels of Erk, Akt, and Stat3 did not change significantly (Figure 3).

### 3.4. DCK or CCNL1 Knockout Does Not Confer Cross-Resistance to Other Drugs

**Figure 4:**
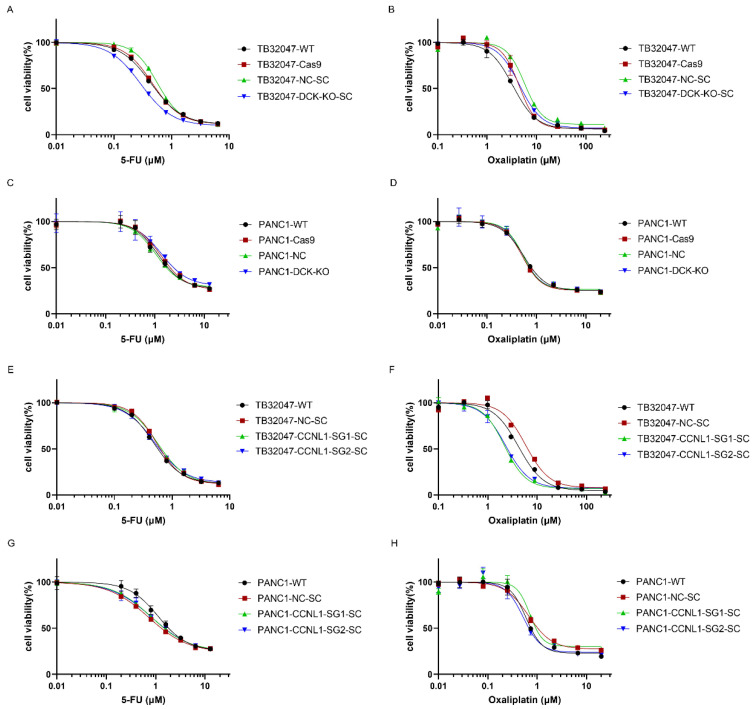
Responses of DCK- and CCNL1-negative cells to 5-FU and oxaliplatin treatment. (**A**,**C**) 5-FU dose–response curves for TB32047 and PANC1 DCK-negative cells. TB32047, but not PANC1 DCK-negative cells, were slightly more sensitive to 5-FU treatment than WT and control cells. (**B**,**D**) Oxaliplatin dose–response curves for TB32047 and PANC1 DCK-negative cells. (**E**,**G**) 5-FU dose–response curves for TB32047 and PANC1 CCNL1-negative cells. (**F**,**H**) Oxaliplatin dose–response curves for TB32047 and PANC1 CCNL1-negative cells. TB32047, but not PANC1 CCNL1-negative cells, showed increased sensitivity to oxaliplatin treatment compared to WT and control cells.

We then investigated the responses of DCK- and CCNL1-negative cells to the commonly used chemotherapeutic drugs 5-FU and oxaliplatin. TB32047, but not PANC1 DCK-negative cells, were slightly more sensitive to 5-FU treatment than WT and the control cells. TB32047, but not PANC1 CCNL1-negative cells, showed an increased sensitivity to oxaliplatin treatment. We observed no significant difference between TB32047 and PANC1 DCK-negative cells in their response to oxaliplatin treatment or between TB32047 and PANC1 CCNL1-negative cells in their response to 5-FU treatment (Figure 4).

The gemcitabine IC50 value for TB32047 and PANC1 WT and NC cells was around 5 nM, for TB32047 CCNL1-negative cells it was nearly double that of WT and NC cells, and for PANC1 CCNL1-negative cells it was around 20 nM. The oxaliplatin IC50 value for TB32047 CCNL1-negative cells was half that of WT cells, and the 5-FU IC50 value for TB32047 DCK-negative cells was 65% that of WT cells.

## 4. Discussion

Chemotherapy is a common treatment for improving pancreatic cancer patient outcomes, and gemcitabine is a widely applied first-line chemotherapy drug. However, drug resistance is a critical issue that considerably impacts the efficacy of chemotherapy. Pooled CRISPR/Cas9 library screening is a powerful tool that facilitates the identification of new targets for limiting drug resistance. We performed genome-wide CRISPR/Cas9 knockout screening in the TB32047 cell line to determine the genes associated with gemcitabine resistance and understand their mechanisms.

We identified several genes that contribute to gemcitabine resistance. The highest-ranking gene was DCK, which is located on chromosome 4q13.3 and codes for the protein deoxycytidine kinase. DCK is a rate-limiting enzyme of the salvage pathway, which is involved in the synthesis of deoxyribonucleotide triphosphates (dNTPs); dNTPs are essential for DNA replication, repair, and tumor growth. Therefore, inhibiting the activity of DCK may inhibit tumor growth [20]. However, this gene is also associated with gemcitabine resistance [21,22]. Deoxycytidine kinase is essential to the drug’s efficacy, as it phosphorylates gemcitabine into gemcitabine monophosphate (dFdCMP), diphosphate (dFdCDP), and triphosphate (dFdCTP), which are cytotoxic [23]. Therefore, the loss of DCK could stop the phosphorylation of gemcitabine and make gemcitabine lose its cytotoxicity. As the highest-ranking gene, DCK is the positive control for our screening and confirms its validity. We successfully knocked out DCK in the TB32047 and PANC1 cell lines using the CRISPR/Cas9 gene-editing system and evaluated the knockout effect by Western blot analysis. We performed an EC50 assay to detect the gemcitabine resistance in DCK-knockout cell lines. The results were consistent with those of other studies [21,22,24]: the loss of DCK induces significant gemcitabine resistance in cells.

CCNL1, located on chromosome 3q25.31, was the second-highest-ranking gene in our list. It is commonly overexpressed in head and neck squamous cell carcinomas (HNSCC) [25] and could play a critical role in their loco-regional progression [26]. CCNL1 may interact with cyclin-dependent kinases (CDKs), participate in pre-mRNA splicing [27], and be inhibited by the CDK-specific inhibitor CDKN1A/p21 [28]. Few studies have considered the role of CCNL1 in pancreatic cancer, and none have examined the connection between CCNL1 and gemcitabine resistance. We used the CRISPR/Cas9 gene-editing system to knockout CCNL1 in TB32047 and PANC1 cell lines. We evaluated the knockout effect using qRT–PCR and sequencing. The EC50 and apoptosis assay results indicated that, compared with WT and NC cells, CCNL1-negative cells were more resistant to gemcitabine treatment, but they were not as resistant as DCK-knockout cells. The knocking-out of CCNL1 hyperactivates survival-associated genes in the MAPK, AKT, and STAT pathways.

The MAPK pathway is essential to cell proliferation, migration, survival, and cancer therapy resistance [29]. Akt is a serine-threonine kinase that likely participates in various biological functions, such as cell proliferation, survival, and apoptosis [30,31]. STAT3 is actively involved in cell growth and survival [32]. Changes to the phosphorylation of Erk [33,34,35], Akt [36,37,38], and STAT3 [39,40] could alter cancer cells’ sensitivity to gemcitabine treatment. In our study, the phosphorylation of Erk, Akt, and STAT3 was hyperactivated after we had knocked out CCNL1 in TB32047 and PANC1 cells, suggesting that the loss of CCNL1 induces resistance to gemcitabine treatment by activating the ERK/AKT/STAT3 survival pathway. However, further investigation into the regulatory mechanisms relating to CCNL1 and the ERK/AKT/STAT3 survival pathway is necessary.

Although the loss of DCK and CCNL1 induced resistance to gemcitabine treatment, we observed different results for the 5-FU and oxaliplatin treatments. TB32047, but not PANC1 DCK-negative cells, were slightly more sensitive to 5-FU treatment, and TB32047, but not PANC1 CCNL1-negative cells, showed increased sensitivity to oxaliplatin treatment. Contrary to the findings for gemcitabine, DCK- and CCNL1-negative cells showed no increased resistance to 5-FU and oxaliplatin treatments. Pancreatic cancer patients may benefit more from combination chemotherapy than long-term gemcitabine treatment.

## 5. Conclusions

We demonstrated that DCK and CCNL1 contribute to gemcitabine resistance in pancreatic cancer by performing genome-wide CRISPR/Cas9 knockout screening in the mouse pancreatic cancer cell line TB32047 under gemcitabine treatment. The knockout of DCK or CCNL1 by the CRISPR/Cas9 system promotes gemcitabine resistance in pancreatic cancer cells. The loss of CCNL1 may activate the ERK/AKT/STAT3 survival pathway, inducing resistance to gemcitabine treatment.

## Figures and Tables

**Table 1:** Sequencing confirmation of mutations in TB32047 and PANC1 CCNL1-knockout single clones.

Cells	Mutation Number	Mutation Size	Mutation Type
TB32047-CCNL1-SG1-SC	1	2 single nucleotides	1 bp mutation 1 bp deletion
TB32047-CCNL1-SG2-SC	2	single nucleotide	deletion
		66 bp	6 bp replaced by 66 bp
PANC1-CCNL1-SG1-SC	2	102 bp	insertion
		359 bp	4 bp replaced by 359 bp
PANC1-CCNL1-SG2-SC	1	single nucleotide	deletion

**Table 2:** IC50 values and 95% CIs (confidence intervals) of chemotherapy drugs in DCK- and CCNL1-knockout cells.

Cells	Gemcitabine (nM)		Oxaliplatin (µM)		5-Flourouracil (µM)	
	IC50	95% CI	IC50	95% CI	IC50	95% CI
TB32047-WT	7.69	7.48–7.91	3.32	3.03–3.64	0.44	0.42–0.46
TB32047-Cas9	8.21	7.95–8.48	4.42	3.92–5.00	0.46	0.45–0.48
TB32047-NC-SC	8.85	8.37–9.35	5.70	4.99–6.48	0.57	0.54–0.60
TB32047-DCK-KO-SC	-	-	4.54	4.28–4.82	0.29	0.28–0.31
PANC1-WT	7.75	6.52–9.18	0.55	0.50–0.61	1.17	0.96–1.46
PANC1-Cas9	9.27	8.22–10.36	0.51	0.47–0.56	1.33	1.17–1.52
PANC1-NC	10.47	9.47–12.55	0.53	0.46–0.61	1.05	0.93–1.20
PANC1-DCK-KO	-	-	0.53	0.44–0.63	1.35	1.01–1.90
TB32047-WT	6.56	5.91–7.29	4.21	3.74–4.76	0.48	0.46–0.50
TB32047-NC-SC	8.42	8.08–8.90	5.88	5.01–6.89	0.54	0.52–0.57
TB32047-CCNL1-SG1-SC	13.41	12.78–14.08	2.17	1.97–2.39	0.56	0.52–0.61
TB32047-CCNL1-SG2-SC	15.53	14.62–16.51	2.30	2.05–2.56	0.50	0.46–0.54
PANC1-WT	4.70	4.00–5.66	0.60	0.53–0.67	1.12	0.93–1.38
PANC1-NC-SC	7.67	6.23–9.60	0.67	0.59–0.76	0.72	0.67–0.77
PANC1-CCNL1-SG1-SC	15.81	14.01–17.89	0.71	0.61–0.87	0.81	0.73–0.89
PANC1-CCNL1-SG2-SC	23.25	19.01–32.83	0.52	0.42–0.64	0.83	0.68–1.05

## Data Availability

Raw data of the CRISPR/Cas9 screening are available in the ENA database, PRJEB51345.

## Supplementary Materials

The following supporting information can be downloaded at: https://www.mdpi.com/article/10.3390/cancers14133152/s1, Figure S1: Knockout of DCK increased gemcitabine resistance in TB32047 and PANC1 cells, Figure S2: Sequencing confirmation of mutation in TB32047 and PANC1 CCNL1-negative cells, Figure S3: Western blot of TB32047 and PANC1 cells transfected with DCK sgRNA or nontargeting control, Figure S4: Western blot of TB32047 and PANC1 cells transfected with CCNL1 sgRNA or nontargeting control, Table S1: TB32047 genome-wide CRISPR/Cas9 gemcitabine screening. Table S2: Quantification of WB data. Intensity of bands as relative light units. No.: Arbitrarily assigned number of the Western blots used for analysis.
