# Supplementary material for: Genome-Wide CRISPR Screening Identifies DCK and CCNL1 as Genes That Contribute to Gemcitabine Resistance in Pancreatic Cancer
Source: Cancers (Basel). 2022 Jun 27;14(13):3152. doi: 10.3390/cancers14133152 (PMC9264918; doi:10.3390/cancers14133152)
Supplement: Supplementary file 1 [file cancers-14-03152-s001.zip › S-fig.pdf]

cancers

MDPI

Supplementary material

Genome-Wide CRISPR Screening Identifies DCK and CCNL1 as Genes that Contribute to Gemcitabine Resistance in Pancreatic Cancer

Hai Yang, Bin Liu, Dongxue Liu, Zhirong Yang, Shuman Zhang, Pengyan Xu, Yuming Xing, Isabella Kutschick, Susanne Pfeffer, Nathalie Britzen-Laurent, Robert Grützmann and Christian Pilarsky

The description of the content for the mouse GeCKO libraries can be found at https://www.addgene.org/pooled- library/zhang-mouse-gecko-v2/

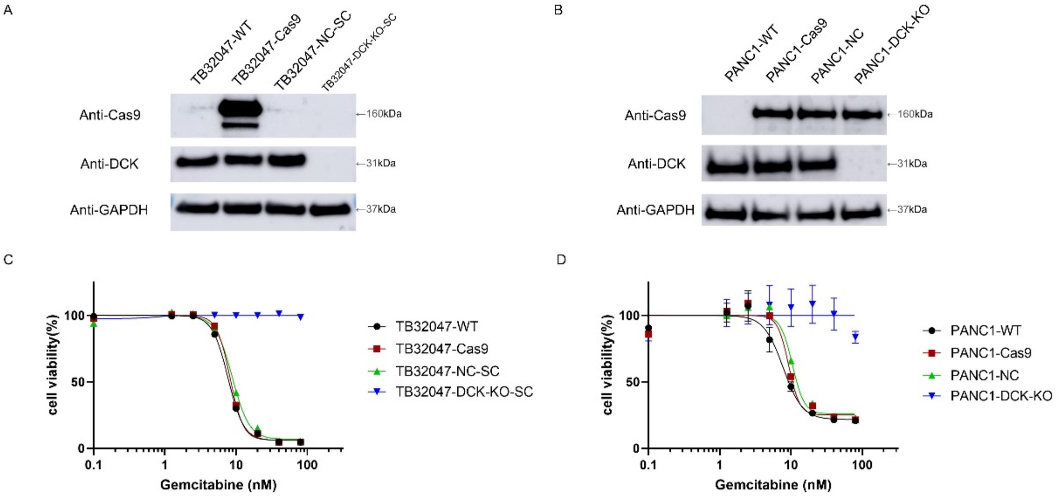

Figure S1. Knockout of DCK increased gemcitabine resistance in TB32047 and PANC1. (A-B) Western blot results show the DCK knocked out in TB32047-DCK-KO-SC PANC1-DCK-KO. (C-D) Gemcitabine dose-response curves for TB32047 and PANC1 cells. The results showed that DCK negative cells were utterly resistant to 80 nM gemcitabine.

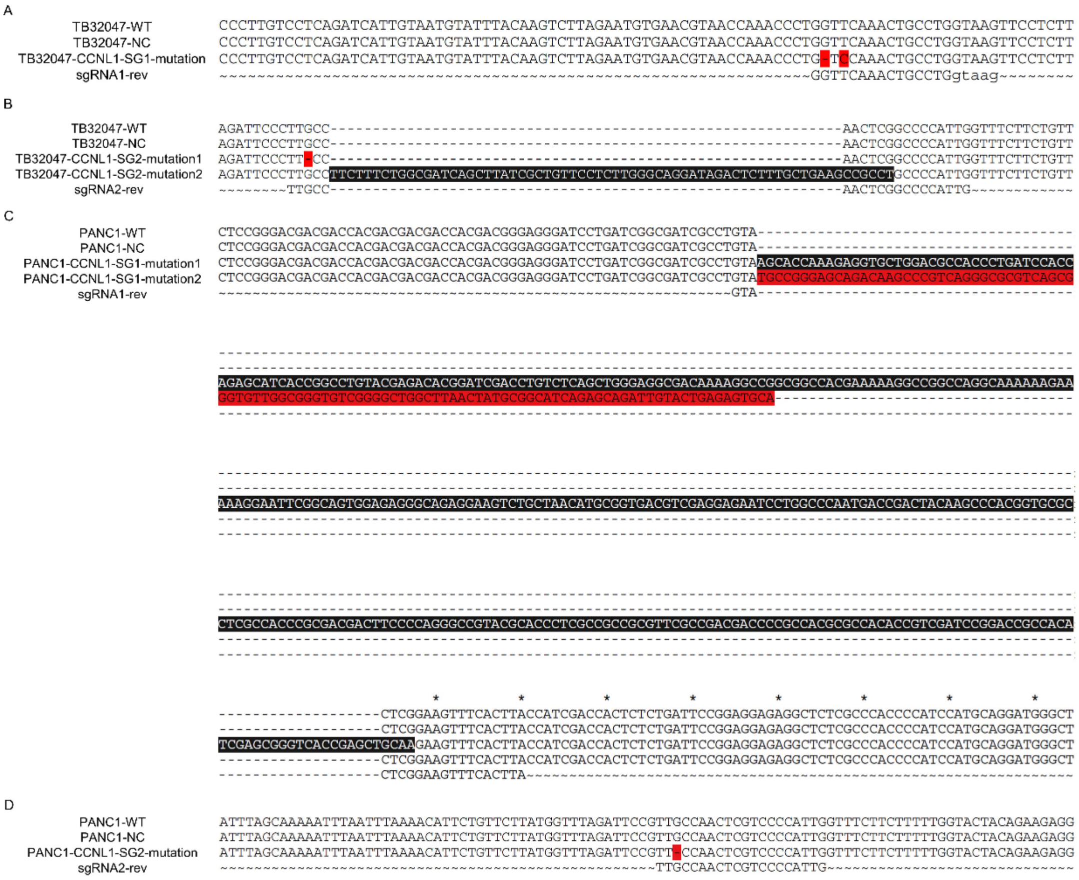

Figure S2. Sequencing confirmation of mutation in TB32047 and PANC1 CCNL1 negative cells. (A) to (D) Sequencing results showing CCNL1 knocked out in TB32047-CCNL1-SG1/2-SC and PANC1-CCNL1-SG1/2-SC. The red highlight area means the insertion, deletion, or replacement of the reference sequence. The black highlight area means a big fragment replacement compared with the reference sequence.

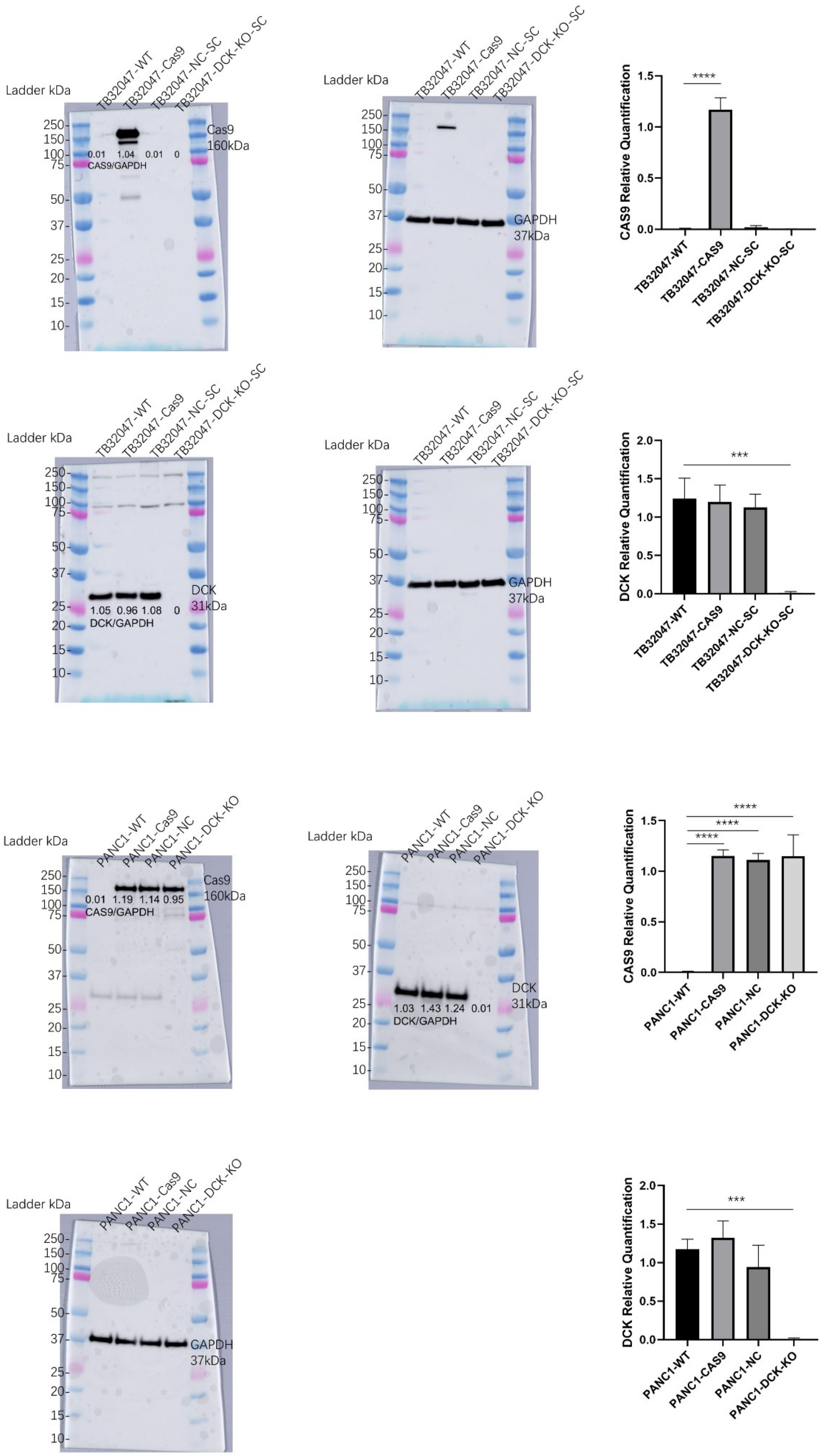

Figure S3. Western blot of TB32047 and PANC1 cells transfected with DCK sgRNA or nontargeting control.

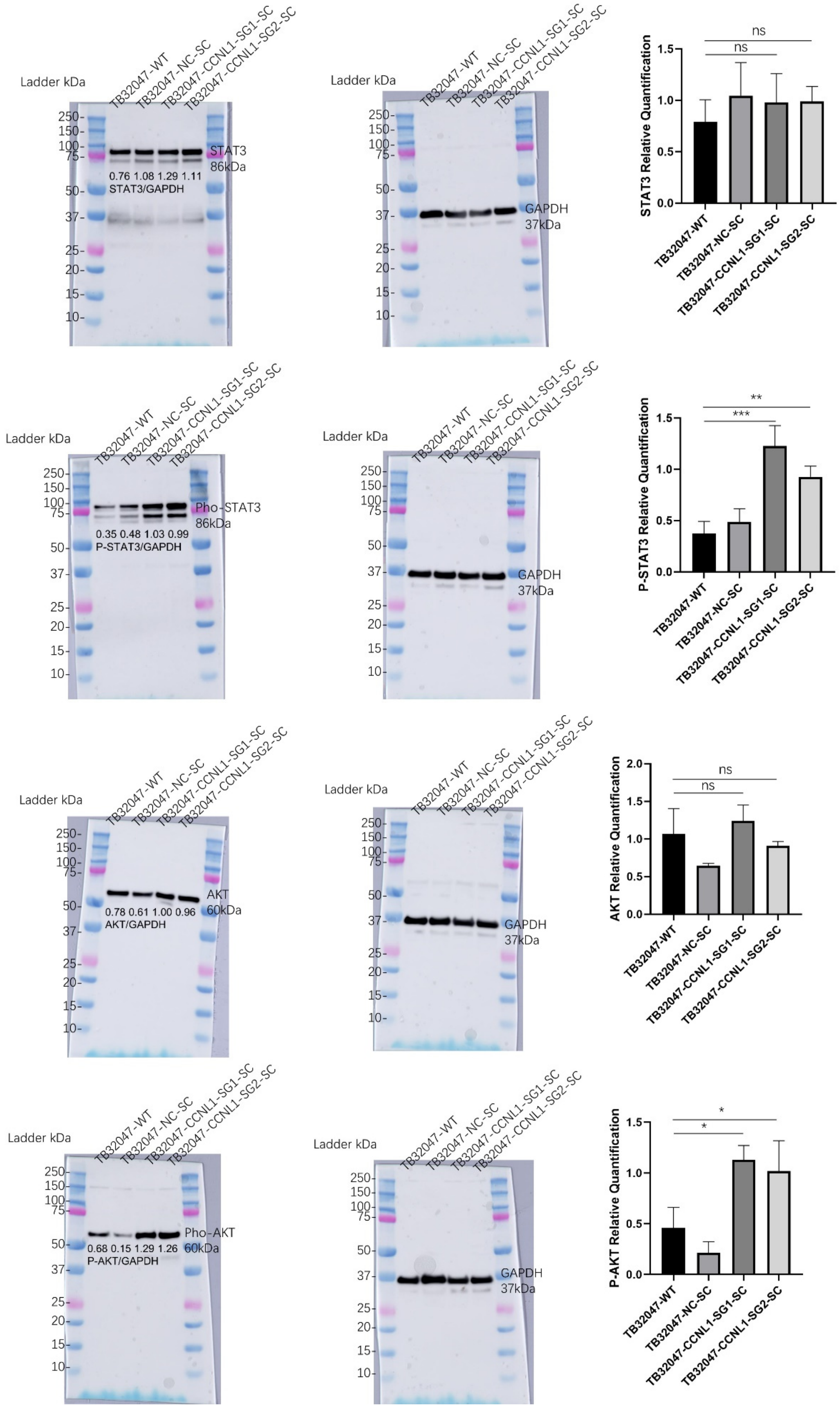

Ladder kDa
TB32047-WT
TB32047-NC-SC
TB32047-CCNL1-SG1-SC
TB32047-CCNL1-SG2-SC
250-
150-
100-
75-
50-
37-
25-
20-
15-
10-
STAT3
86kDa
0.76 1.08 1.29 1.11
STAT3/GAPDH
GAPDH
37kDa
STAT3 Relative Quantification
ns
ns
Pho-STAT3
86kDa
0.35 0.48 1.03 0.99
P-STAT3/GAPDH
P-STAT3 Relative Quantification
**
***
AKT
60kDa
0.78 0.61 1.00 0.96
AKT/GAPDH
AKT Relative Quantification
Pho-AKT
60kDa
0.68 0.15 1.29 1.26
P-AKT/GAPDH
P-AKT Relative Quantification
*
*

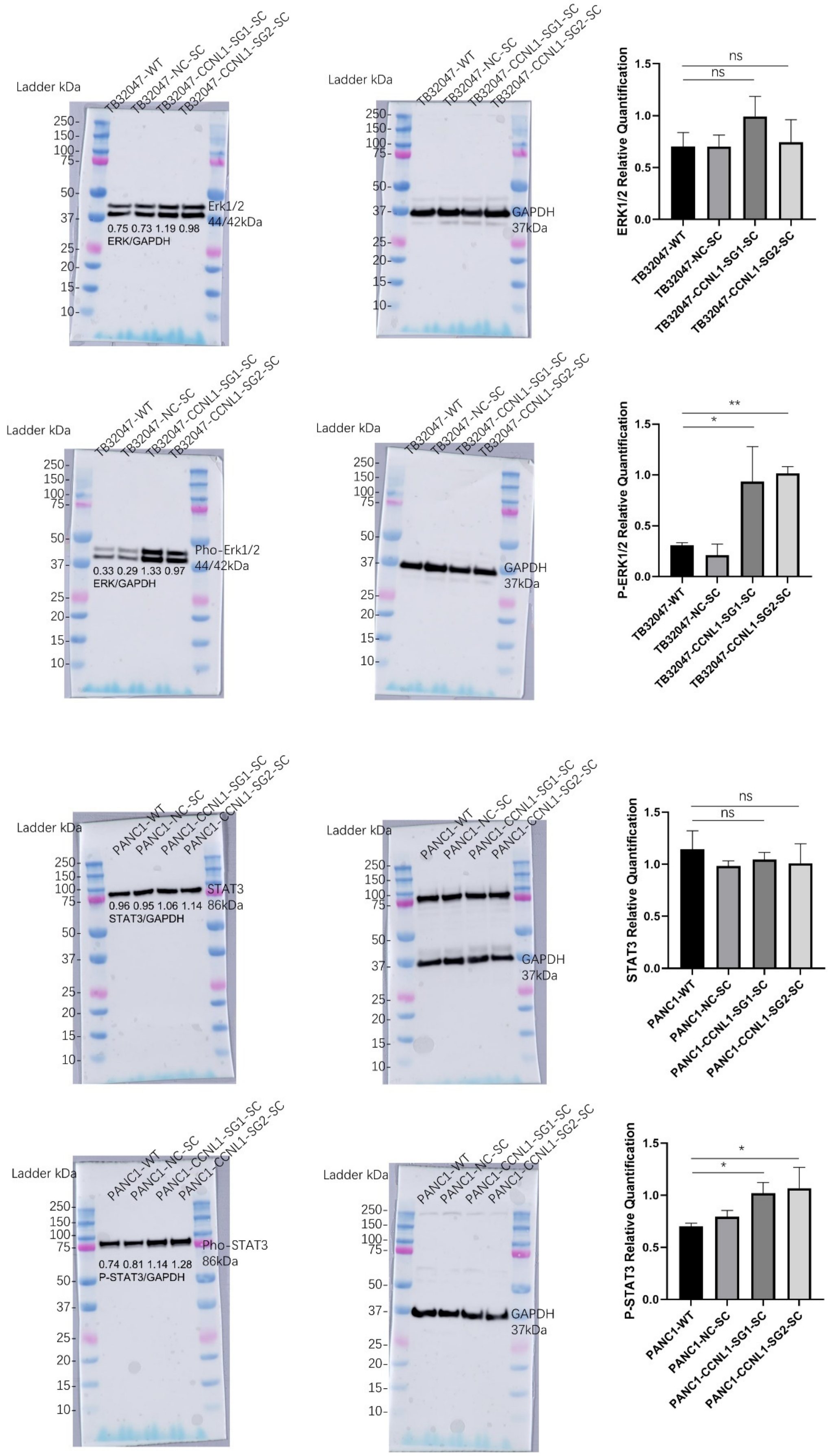

Ladder kDa
TB32047-WT
TB32047-NC-SC
TB32047-CCNL1-SG1-SC
TB32047-CCNL1-SG2-SC
250
150
100
75
50
37
25
20
15
10
Erk1/2
44/42kDa
0.75 0.73 1.19 0.98
ERK/GAPDH
GAPDH
37kDa
ERK1/2 Relative Quantification
1.5
1.0
0.5
0.0
ns
ns
Pho-Erk1/2
44/42kDa
0.33 0.29 1.33 0.97
ERK/GAPDH
P-ERK1/2 Relative Quantification
**
*
PANC1-WT
PANC1-NC-SC
PANC1-CCNL1-SG1-SC
PANC1-CCNL1-SG2-SC
STAT3
86kDa
0.96 0.95 1.06 1.14
STAT3/GAPDH
STAT3 Relative Quantification
Pho-STAT3
86kDa
0.74 0.81 1.14 1.28
P-STAT3/GAPDH
P-STAT3 Relative Quantification

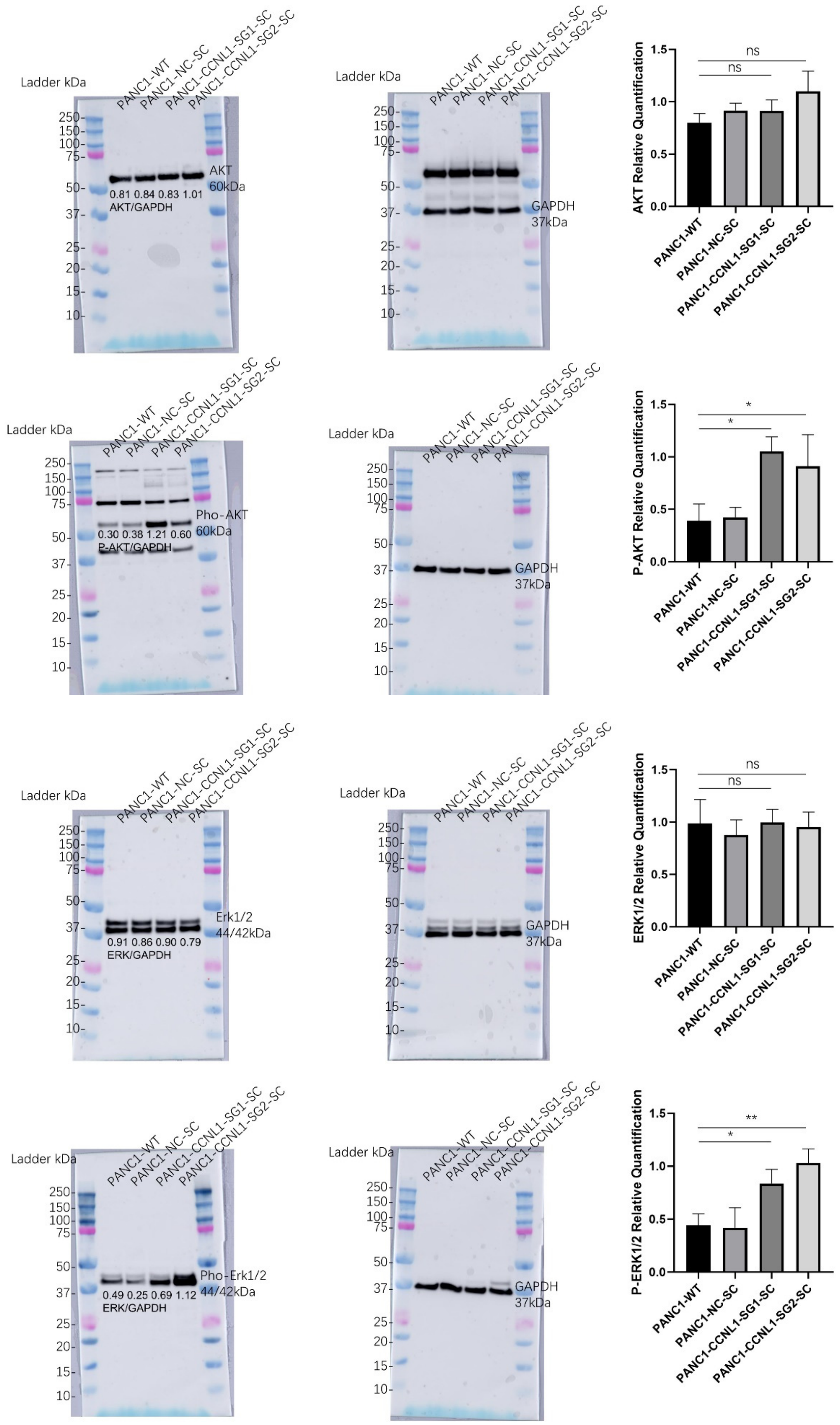

Figure S4. Western blot of TB32047 and PANC1 cells transfected with CCNL1 sgRNA or nontargeting control.
