# Supplementary material for: Genome-Wide CRISPR Screening Identifies DCK and CCNL1 as Genes That Contribute to Gemcitabine Resistance in Pancreatic Cancer
Source: Cancers (Basel). 2022 Jun 27;14(13):3152. doi: 10.3390/cancers14133152 (PMC9264918; doi:10.3390/cancers14133152)
Supplement: Supplementary file 1 [file cancers-14-03152-s001.zip › Table S2.pdf]

cancers

MDPI

Table S2. Quantification of WB data. Intensity of bands as relative light units. No.: Arbitrarily assigned number of the Western blots used for analysis. .

CAS9	TB32047-WT	TB32047-CAS9	TB32047-NC-SC	TB32047-DCK-KO-SC
No. 1	0,00983	1,042459	0,010451	0,003478
No. 2	0,004421	1,275084	0,009218	0,003601
No. 3	0,008476	1,186867	0,039981	0,004309
DCK	TB32047-WT	TB32047-CAS9	TB32047-NC-SC	TB32047-DCK-KO-SC
No. 1	1,045699	0,955817	1,083641	0,001901
No. 2	1,129337	1,391768	1,316326	0,005466
No. 3	1,54556	1,239821	0,979504	0,030932
CAS9	PANC1-WT	PANC1-CAS9	PANC1-NC	PANC1-DCK-KO
No. 1	0,008865	1,192953	1,13576	0,945673
No. 2	0,012327	1,083415	1,16051	1,368772
No. 3	0,00765	1,180473	1,03543	1,13165
DCK	PANC1-WT	PANC1-CAS9	PANC1-NC	PANC1-DCK-KO
No. 1	1,029258	1,427191	1,238973	0,010873
No. 2	1,238103	1,471721	0,91272	0,02302
No. 3	1,261114	1,069495	0,673947	0,003354
STAT3	TB32047-WT	TB32047-NC-SC	TB32047-CCNL1-SG1-SC	TB32047-CCNL1-SG2-SC
No. 1	0,756029	1,081582	1,291026	1,109283
No. 2	1,020306	1,348033	0,8987	0,828383
No. 3	0,591612	0,705559	0,747677	1,031253
P-STAT3	TB32047-WT	TB32047-NC-SC	TB32047-CCNL1-SG1-SC	TB32047-CCNL1-SG2-SC
No. 1	0,354066	0,47775	1,033604	0,991921
No. 2	0,50051	0,620335	1,43211	0,979588
No. 3	0,266855	0,36338	1,213576	0,803833
AKT	TB32047-WT	TB32047-NC-SC	TB32047-CCNL1-SG1-SC	TB32047-CCNL1-SG2-SC
No. 1	0,776362	0,606362	1,000661	0,959757
No. 2	1,436468	0,658487	1,332361	0,849343
No. 3	0,979549	0,668946	1,389699	0,920197
P-AKT	TB32047-WT	TB32047-NC-SC	TB32047-CCNL1-SG1-SC	TB32047-CCNL1-SG2-SC
No. 1	0,679691	0,147145	1,29252	1,258378
No. 2	0,413553	0,339532	1,037996	1,114887
No. 3	0,27963	0,156495	1,056761	0,684011
ERK	TB32047-WT	TB32047-NC-SC	TB32047-CCNL1-SG1-SC	TB32047-CCNL1-SG2-SC
No. 1	0,748531	0,731382	1,188474	0,983086
No. 2	0,809349	0,577329	0,989491	0,693631
No. 3	0,549911	0,795382	0,799871	0,556347
P-ERK	TB32047-WT	TB32047-NC-SC	TB32047-CCNL1-SG1-SC	TB32047-CCNL1-SG2-SC
No. 1	0,329998	0,290356	1,326615	0,972017
No. 2	0,281148	0,255843	0,797946	1,091565
No. 3	0,313414	0,086834	0,683238	0,984748
STAT3	PANC1-WT	PANC1-NC-SC	PANC1-CCNL1-SG1-SC	PANC1-CCNL1-SG2-SC
No. 1	0,962239	0,951234	1,060122	1,136721
No. 2	1,153772	1,039694	0,970515	0,795047
No. 3	1,31628	0,962304	1,104736	1,097922
P-STAT3	PANC1-WT	PANC1-NC-SC	PANC1-CCNL1-SG1-SC	PANC1-CCNL1-SG2-SC
No. 1	0,736209	0,808726	1,136678	1,278955
No. 2	0,690895	0,729181	0,978325	0,878905
No. 3	0,67754	0,846206	0,945614	1,04538
AKT	PANC1-WT	PANC1-NC-SC	PANC1-CCNL1-SG1-SC	PANC1-CCNL1-SG2-SC
No. 1	0,813737	0,841157	0,834016	1,006746
No. 2	0,704142	0,985394	0,871826	0,971626
No. 3	0,87905	0,919118	1,0315	1,320173
P-AKT	PANC1-WT	PANC1-NC-SC	PANC1-CCNL1-SG1-SC	PANC1-CCNL1-SG2-SC
No. 1	0,300253	0,380862	1,211282	0,597457
No. 2	0,575888	0,531696	0,978595	0,942167
No. 3	0,293938	0,358224	0,963987	1,193162
ERK	PANC1-WT	PANC1-NC-SC	PANC1-CCNL1-SG1-SC	PANC1-CCNL1-SG2-SC
No. 1	0,914579	0,855221	0,899002	0,789124
No. 2	0,805783	0,743637	0,952951	1,040967

No. 3	1,243113	1,030997	1,137792	1,032207
P-ERK	PANC1-WT	PANC1-NC-SC	PANC1-CCNL1-SG1-SC	PANC1-CCNL1-SG2-SC
No. 1	0,487105	0,250575	0,685968	1,116594
No. 2	0,32009	0,383145	0,939275	1,097394
No. 3	0,521789	0,62507	0,88506	0,877128
